# The Obtunding of Sensitive Dentine

**Published:** 1891-04

**Authors:** George F. Eames


ARTICLE II.

### THE OBTUNDING OF SENSITIVE DENTINE.

BY GEORGE F. EAMES, M. D., D. D. S.

The dentist who has just used an excavator in removing a little superficially decayed dentine finds an immediate response from the patient, who informs the operator that he has "struck the nerve." The dentist kindly explains that it is only "sensitive dentine," at a considerable distance from the nerve itself. If asked, "what *is* sensitive dentine?" the practitioner will say, if well read in all the modern literature pertaining to this subject, "I do not know; I can only designate it by certain phenomena consequent upon irritation of this structure. We all know that it is often sensitive or painful when touched, and we name the condition from this symptom alone, because we have no real knowledge as to the *modus operandi* or mechanism by which pain is induced, or of the peculiar condition of the parts involved which permit these phenomena."

In recent years pain obtundents and local anæsthetics have been introduced in great profusion, while there is a scarcity of literature which considers the physiological and pathological basis upon which anæsthetic agents may be used. This indicates that the general practice is largely empirical, and this impedes the progress of the profession.

It is time now to ask why the obtundent obtunds and how it does it. Allow me, if you please, to refer to the anatomy and physiology of tooth structure only in sufficient measure to be of assistance in more clearly elucidating the subject. We are to speak principally of the dentine which forms the main bulk of the tooth, and within which is a canal or canals with the largest diameter at the pulp. At the periphery of the pulp and the dentine is a layer of odontoblastic cells which send prolongations into the dentinal tubuli.

This description is, in substance, I believe, generally accepted, although, like other anatomical structures that are microscopic, it may be questioned. Magitot, for instance, denies the existence of these odontoblastic cells, holding that the dentinal fibril is a continuation of a layer of reticulate cells which lie beneath the odontoblasts, while Klein maintains that the only office of the odontoblast is in the formation of the dentine matrix, the dentinal fibril being a prolongation of cells originating between the odontoblasts.

All must agree, however, in the existence of a protoplasmic material throughout the entire structure of the dentine, giving it both nourishment and sensation. We say sensation; *why* do we say it? Is the dentinal fibril capable of transmitting sensation? Let us see. The functions of the dentinal fibril, the various influences that may affect it, its relation to the tooth-pulp, may well form the principal subject-matter for our study in sensitive dentine, for it seems only reasonable that, whatever may be the method of conducting painful sensations from the dentine to the brain, this living matter in the dentine must be the *medium* through which it reaches the pulp. The sensation of pain having reached the pulp, we readily see how it may be conveyed to the brain by reason of the well-known function of the sensory nerves; but the dentine has no nerves; this protoplasmic material occupying the dentinal tubuli has not been shown to be nerve structure. How, then, may it be the medium or have the power of transferring sensations?

We have already noticed the very intimate relations existing between the odontoblastic prolongations or dentinal fibrils and the pulp. Let us now notice some of the characteristics of this so-called dentinal fibril. For this purpose we have recourse to some of the simplest forms of life, and from them receive valuable knowledge, as Paget so admirably writes: "The highest laws of our science are expressed in the simplest terms in the lives of the lowest orders of creation." These odontoblastic cells forming the dentinal fibril seem to consist of simple masses of protoplasm, of which the amœbæ and the leucocytes are good examples, all possessing similar characteristics, so that we may draw reasonable conclusions from them. The amœbæ may be easily observed under the microscope. We find that it is capable of moving with extreme slowness from place to place; that it is sensitive to mechanical and chemical irritation, as shown by the change in its movements and in its form. It is especially sensitive to thermal changes. If the temperature be raised to 95 or 100° F., the movements are arrested, but if this amount of heat be not sustained too long, the amœba will resume its power of moving. The same thing occurs when the temperature is reduced to the freezing point. If the temperature is reduced below the freezing point or raised above 105° F., the movements entirely cease and are not resumed on raising or lowering the temperature again; in other words, there is cessation of function—the amœba is dead. This is the effect of heat and cold upon protoplasm, as shown in the amœba, the leucocyte, and, with possible modification, in the dentinal fibril.

These results suggest the possibility of painful sensations being transmitted to the pulp and thence to the brain through the agency of the fibril. They also suggest the possibility of injury to the pulp by means of hot or cold applications to cavities in the teeth. If this theory be true, then in every cut into the dentine with the excavator we are wounding living tissue, and this irritation is converted into pain when it reaches the pulp.

But my main purpose in calling your attention to the anatomy and physiology of the dentine is that we might better understand the effect of heat or cold as applied to sensitive dentine. Heat is applied in various ways, dry or moist, but one of the recent methods of using moist heat is that in which a jet of steam is applied to the cavity; others consist in the use of a spray from a very volatile liquid like chloride of methyl, bromide of ethyl, or ether. In the use of these agents, as well as steam, the heat or cold is intense, and it seems to me that, in making use of such extremes of heat and cold, we may be expecting too much of the vitality of the dental pulp. The protoplasmic cell, identical in its nature with the dentinal fibril, is destroyed at a temperature above 105° F. or below 32° F.

The dentinal fibril being in such close connection with the pulp, and perhaps through it in some way fortified with an extra amount of vital force, or resisting power, may be able to recover after the application of a greater amount of heat or cold than a portion of isolated protoplasm. However this may be in the light of the foregoing statements, I am bound to say that I believe there is danger in the general use of such extremes of heat and cold as are produced by the spray of ether or chloride of methyl.

I am fully aware that these agents are used in a large majority of cases, as I have used them myself without perceptible injurious results at the time, and I believe that what has been written advocating these remedies is in the right direction, but I do not think sufficient time has elapsed to enable us to judge correctly. An application of steam two seconds longer in the case of one patient than in another may be enough to make the difference between success and failure. It seems to me that such nice adapt ation to the temperament of an individual cannot always be made by ordinary mortals.

Now, I am sure that we can make use of heat or cold in sensitive dentine without injurious results, and in this way: By using an apparatus which will indicate the temperature; in other words, by knowing the dose to be administered. The method which I wish to commend, and which I believe can be used in the greatest number of ways, is the use of warm air at a certain definite degree. If we are to be guided at all by our observations of the effect of changes in temperature on the amœba, we shall not raise the heat above 110° F. or reduce it below the freezing point. I am sure that I have accomplished much with this degree of heat, although I do not pretend in any dogmatic sense to fix the border line beyond which we cannot use heat or cold with safety.

I have recently been using Codman & Shurtleff's air-compressor, and have had several appliances made to connect with it, heating the air, still under pressure, and conveying it to the mouth, and by means of a valve and fine tube having it under full control. My last apparatus was an air-tight brass tank containing a thermometer, which indicates correctly the temperature of air *in the tank*, but very hot air in passing through six feet of rubber tubing will become cold.

I am experimenting with a much smaller instrument, which can be placed on the bracket table and brought near the patient.

Air, under pressure, made warm at will, may be made to serve many useful purposes besides obtunding pain. It is the very best means of producing a spray from any liquid, and there are many uses for the spray. Hot medicated air may be driven with great force if desired into a root canal, or used as a chip-blower, or to hasten the hardening of modeling composition when used for impressions of the mouth, to soften gutta-percha fillings or crown-settings, etc.

Permit me now, if you please, to indicate something in the line of treatment according to my view of the situation. We may find a condition of general or local nerve irritability, or both. I sometimes treat systemically, using laxatives if needed, followed by such sedatives as sodium bromide, Jamaica, dogwood, or aconite, avoiding the menstrual period when possible, and giving morning appointments as a rule.

A combination of kindness, patience, mesmerism, hypnotism, Christian science, mental healing, magnetism, sharp instruments and a steady hand is always on the shelf for use, and I see no antagonism or incompatibility between the ingredients. For the local and the general condition in excessively sensitive dentine, the inhalation of ether has been with me a decided success. The patient usually holds the napkin and inhales until the first effects are produced, stopping short of unconsciousness. The odor is an objection, of course. I do not use it often, but when I do it is to me the king of remedies. I have frequently used nitrous oxide for this purpose with much satisfaction. But cocaine is the remedy that I use most, and I believe it to be the safest and most efficient local anæsthetic that we have at our command to-day. This is the way I use this agent and succeed : After the cavity is dry I make it dryer by the use of warm air; the dehydration of the tubules and the raised temperature not only lessen the sensibility, but also provide for the absorption of any medicament that may be placed in the cavity. Alcohol is also used for drying, alternating with the warm air until I think it sufficient for the purpose; it is then ready for the cocaine. I formerly made an alcoholic solution, but believe it to be better practice to use the cocaine and alcohol separately.

Our studies in experimental therapeutics show that alcohol is absorbed very slowly, if at all, while acidulous and chloroformic solutions are absorbed with facility. I therefore have used chloroform, alternating with an aqueous solution of cocaine, although I now make my solution of cocaine in chloroform alone. The dentinal tubules being deprived of moisture by means of alcohol and warm air, now readily drink up a chloroformic solution of cocaine.

I have used this method with the above modification with decided success. I think it is believed by the profession generally that cocaine is of little value in sensitive dentine, and I had reached this conclusion myself; but a physician once insisted upon my using it in his tooth, and he had so much faith in its efficacy, from his experience with it in the soft tissues, that I made a thorough application with fair success; later I became interested in experimental therapeutics, resulting in the use of cocaine as I now use it. The only obstacle to its application in and around the teeth is the difficulty of securing absorption. If you once cause it to reach the spot where you want it to act, it will anæsthetize every time it is applied.—*International Dental Journal.*

---

ARTICLE III.

### THE TREATMENT OF DEAD TEETH.

